# Supplementary material for: Drug repurposing for aging research using model organisms
Source: Aging Cell. 2017 Jun 16;16(5):1006–15. doi: 10.1111/acel.12626 (PMC5595691; doi:10.1111/acel.12626)
Supplement: Supplementary file 7 — Data S1 Zip‐Archive of all report cards. [file ACEL-16-1006-s007.zip › RC_NEQ.pdf]

NEQ

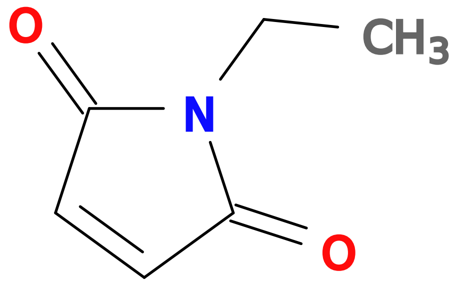

Database identifiers

ChEMBLCompound	CHEMBL8211
DrugBank	DB02967
CHEBI	44485
ZINC	ZINC00901367
eMolecules	475873

Ranking

	Rank	Score
Drosophila	538/697	0.217
C. elegans	311/591	0.187

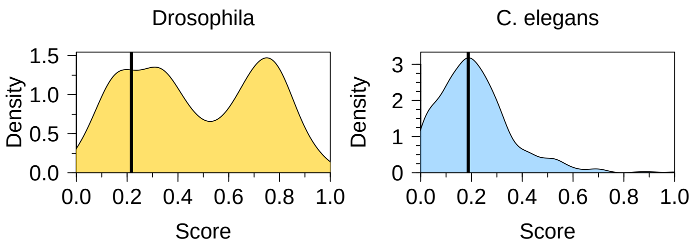

	Ageing implication	Domain conservation	Binding site conservation	Binding affinity	Bioavailability	Lipinski	Promiscuity	Purchasability	Drug approval	**Total**
Drosophila	0.203	0.89	0.652	0.401	(0.9)	0.0	-0.0	0.1	0.075	0.217
C. elegans	0.203	0.886	0.608	0.401	0.296	0.0	-0.0	0.1	0.075	0.187

Names

- **N-ethylmaleimide**
- NEM

Roles

EC 2.7.1.1 (hexokinase) inhibitor, EC 1.3.1.8 [acyl-CoA dehydrogenase (NADP(+))] inhibitor, EC 2.1.1.122 [(S)-tetrahydroprotoberberine N-methyltransferase] inhibitor

Status

Approved drug (according to ChEMBL)	No
Classification (according to DrugBank)	experimental
Number of Rule of 5 violations	0
Binding affinity to original target in log units (RF-Score prediction)	4.6
Burns *C. elegans* bioavailability prediction	-0.49

Compound Target Characteristics

Proliferating cell nuclear antigen

Best gene implication in ageing for this target family came from gene P12004 via mapping the annotation from Ensembl ENSG00000132646 via mapping the annotation from EntrezGene 5111 via mapping the annotation from GenAgeHuman 0113 annotated in GenAge release 17.

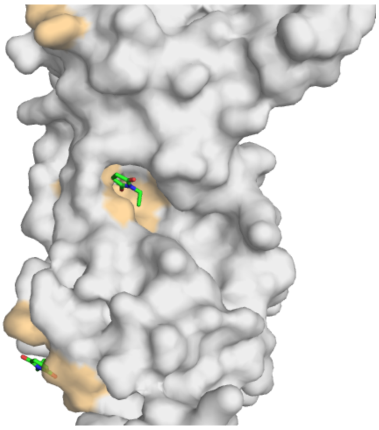

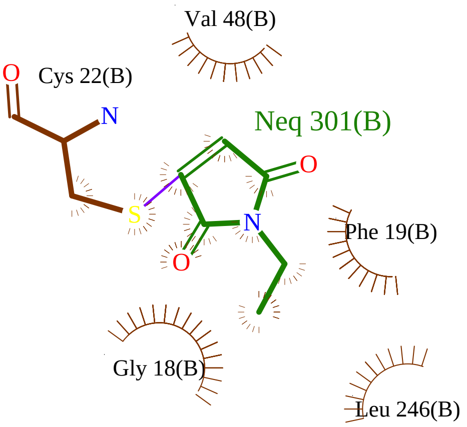

protein	amino acids contacts (binding site)
PDB:3v61:chainB:P15873	G F C V K I C Y Q L D L F
PDB:3v62:chainB:P15873	G F C V K I C Y Q L D L F
sp:P12004:PCNA_HUMAN	A L L V K I C Y H I F L Y
sp:P04961:PCNA_RAT	A L L V K I C Y H I F L Y
tr:Q542J9:Q542J9_MOUSE	A L L V K I C Y H I F L Y
sp:P17918:PCNA_MOUSE	A L L V K I C Y H I F L Y
sp:P17917:PCNA_DROME	A I L V K I C Y Q F A I Y
sp:O02115:PCNA_CAEEL	S I L V K A C V T F Q L F
sp:P15873:PCNA_YEAST	G F C V K I C Y Q L D L F

	whole protein		domain-based		contact-based	
protein	ident	simil	ident	simil	ident	simil
PDB:3v61:chainB:P15873	1.0	1.0	1.0	1.0	1.0	1.0
PDB:3v62:chainB:P15873	1.0	1.0	1.0	1.0	1.0	1.0
sp:P12004:PCNA_HUMAN	0.35	0.8	0.35	0.81	0.46	0.61
sp:P04961:PCNA_RAT	0.35	0.8	0.35	0.8	0.46	0.61
tr:Q542J9:Q542J9_MOUSE	0.35	0.8	0.35	0.8	0.46	0.61
sp:P17918:PCNA_MOUSE	0.35	0.8	0.35	0.8	0.46	0.61
sp:P17917:PCNA_DROME	0.36	0.81	0.36	0.81	0.46	0.65
sp:O02115:PCNA_CAEEL	0.4	0.8	0.4	0.81	0.38	0.61
sp:P15873:PCNA_YEAST	1.0	1.0	1.0	1.0	1.0	1.0

mus209 (FBgn0005655) associated phenotypes

DNA repair defective, cold sensitive, endomitotic cell cycle defective, heat sensitive, lethal - all die before end of pupal stage, neuroanatomy defective, oxidative stress response defective, some die during pupal stage, suppressor of variegation

(Information from FlyBase)

PCNA (UniProt:P17917) annotation

Function: This protein is an auxiliary protein of DNA polymerase delta and is involved in the control of eukaryotic DNA replication by increasing the polymerase's processibility during elongation of the leading strand.

Subunit: Homotrimer. Forms a complex with activator 1 heteropentamer in the presence of ATP (By similarity). Interacts with E2f. (, PubMed:19081076).

Subcellular location: Nucleus (PubMed:9310333). Note=Colocalizes with crm in polytene nuclei during embryogenesis.

Tissue specificity: Expressed at high levels in adult ovary. (PubMed:1968224).

Developmental stage: Expressed maternally and zygotically. Expressed at high levels in unfertilized eggs and during early stages of embryogenesis. Low expression detected during late embryogenesis, in second and third instar larvae and in adult flies. (PubMed:1968224).

Disruption phenotype: Mutant flies show temperature-sensitive lethality, hypersensitivity to DNA-damaging agents such as ionizing radiation and methyl methanesulfonate, suppression of position-effect variegation and female sterility. (PubMed:7907981).

(Information from UniProt)

pcn-1 (WBGene00003955) associated phenotypes

lethal, sterile

(Information from WormBase)

pcn-1 (UniProt:O02115) annotation

Function: This protein is an auxiliary protein of DNA polymerase delta and is involved in the control of eukaryotic DNA replication by increasing the polymerase's processibility during elongation of the leading strand.

Subunit: Homotrimer. Forms a complex with activator 1 heteropentamer in the presence of ATP (By similarity).

Subcellular location: Nucleus.

(Information from UniProt)
